# The flow of an Eyring Powell Nanofluid in a porous peristaltic channel through a porous medium

**DOI:** 10.1038/s41598-023-36136-x

**Published:** 2023-06-15

**Authors:** Sohail Nadeem, Aiman Mushtaq, Jehad Alzabut, Hassan Ali Ghazwani, Sayed M. Eldin

**Affiliations:** 1 Department of Mathematics, Quaid-I-Azam University, Islamabad, 44000 Pakistan; 2 Department of Mathematics and Sciences, Prince Sultan University, 11586 Riyadh, Saudi Arabia; 3 Department of Mathematics, Wenzhou University, Wenzhou, 325035 China; 4 Department of Industrial Engineering, OSTIM Technical University, 06374 Ankara, Turkey; 5 Department of Mechanical Engineering, Faculty of Engineering, Jazan University, P.O. Box 45124, Jazan, Kingdom of Saudi Arabia; 6 Center of Research, Faculty of Engineering, Future University in Egypt, New Cairo, 11835 Egypt

**Keywords:** Biotechnology, Physiology, Mathematics and computing, Physics

## Abstract

In a porous medium, we have examined sinusoidal two-dimensional transport enclosed porous peristaltic boundaries having an Eyring Powell fluid with a water containing $\text{Al}_{2}{\text{O}}_{3}$. The determining momentum and temperature equations are solved semi-analytically by using regular perturbation method and Mathematica. In present research only free pumping case and small amplitude ratio is studied. Mathematical and pictorial consequences are investigated for distinct physical parameters of interest like porosity, viscosity, volume fraction and permeability to check the effects of flow velocity and temperature.

## Introduction

Peristalsis is a transport generated by the wave-like contraction and expansion of a duct-containing fluid. If there is no peristaltic movement then food bolus cannot be pushed through the digestive tract, urine cannot be moved through the urinary duct and spermatozoon, ovum, embryos and eggs cannot be moved and transferred into a reproductive duct. A person faces diarrhea or constipation in the absence of peristaltic motion. Also, the movement of lymph through lymphatic vessels and the motion of capillaries, arterioles and venules is peristaltic. Peristaltic pumping also acts as a cleansing agent that removes bacteria and gas and controls bacterial growth in large intestines. The peristaltic mechanism is applicable in engineering and biomathematics like the design of finger pumps and roller pumps, dialysis and blood pump machines.

The First theoretical and experimental study on the topic of peristalsis in which the author derives the relation between motion of fluid and the amplitude ratio of the peristaltic transport has been done “Peristaltic transport”, by Fung and Yih^1^ and further work on this topic is proceeded by Latham^2^ in detail as *Fluid motions in Peristaltic Pump* in MIT-Press, Cambridge. There are many investigations studied mathematically for Newtonian and Non-Newtonian fluids. Since there is a vast use of fluids like water, air, glue, paint and blood in our daily life, this domain of research has sought the attention of researchers.

Jaffrin and Shapiro have done many investigations like^3^ “Peristaltic transport” in which he uses a sinusoidal channel to describe the transport of mass and finds the mean average velocity of the transport and^4^ he study about arbitrary Reynold number, wave number and amplitude ratio for peristaltic pumping. Srivastava et al.^5^ worked on the perturbation solutions of peristaltic flow in a channel filled with Newtonian fluid for a small amplitude ratio. Husseny et al*.*^6^ have looked into the Effects of porous boundary walls on peristaltic walls through a porous medium”. “The flow of Maxwell fluids in porous media and peristaltic domain” is examined by Whitaker et al*.*^7^. Moreover, Al-Arabi et al*.*^8^ also show their interest in the domain of peristaltic flow and explored Non-linear peristaltic channel of Magnetohydrodynamic flow. Further “Peristaltic Flow of a Maxwell fluid Mode through Porous Boundaries in a Porous Medium^9^ and Corrugated walls analysis in microchannels through porous medium under Electromagnetohydrodynamic (EMHD) effects^10^ has been examined by Nadeem et al*.* The peristaltic channel has scientific contributions in industrial and biomedical applications and drug delivery.

Eyring Powell fluid is a non-Newtonian viscoelastic fluid which has been studied by Eyring Powell in 1944. Although Eyring Powell fluid is discussed by some researchers but still it is not very common in the area of research. Akbar and Nadeem^10^ has been studied the characteristics of heating scheme and mass transfer on the peristaltic flow for an Eyring-Powell fluid in an endoscope. Moreover, Khan^11^ analyzed the Analysis of eyring–powell fluid flow used as a coating material for wire with variable viscosity effect along with thermal radiation and joule heating. Also Riaz and Ellahi studied Role of hybrid nanoparticles in thermal performance of peristaltic flow of Eyring–Powell fluid model^12^. In this subclass of fluid which deals with Eyring Powell fluid there is not much research but all research in this subclass is not mentioned here. The Eyring Powell fluid has significant impact on the field of fluid mechanics like in viscosity prediction, reaction kinematics, molecular diffusion, polymer science and lubrication technology.

The Nanoparticles like metals, oxides, carbides, $\text{Al}_{2}{\text{O}}_{3}$ or carbon nanotubes are mostly used in Nanofluids. These are mostly used as coolants and have many applications worldwide. Also, Nanofluids have a wide range of literature in ‘fluid mechanics’ as Riaz and Ellahi^11^ studied the “Role of hybrid nanoparticles in the thermal performance of the peristaltic flow of Eyring–Powell fluid”. Moreover, McGrail et al*.*^13^ studied Metal–organic heat carrier nanofluids. Transport properties of non-Newtonian nanofluids and applications is studied by the Sivaraj et al*.*^14^. Also, Naveen Kumar analyzed non-Newtonian hybrid nanofluid flow over vertically upward/downward moving rotating disk in a Darcy–Forchheimer porous medium^15^.

Fluid for a permeable medium has a broad scope in filtration, ground water flow, biomedical applications and importance in engineering and fluid mechanics. In our daily life, permeable media includes wood, foam, and rocks. Industrial and domestic applications of a thin permeable layer are cells, batteries, filters and printing papers. Permeable media had drawn the attention of many researchers such as the “Effects of porous boundaries on peristaltic transport through a porous medium”^6^ is studied by Shehawey and Husseny. Nonlinear peristaltic transport of MHD flow through a porous medium was investigated by Mekheimer^8^. Further, Peristaltic flow of a Maxwell fluid in a channel with compliant walls is discussed and analyzed by Hayat^17^. Also Alame^18^ take part in the research of this field by using uniform magnetic field in the channel having non-Newtonian Fluid with heat and mass transfer through a porous medium. Further, Peristaltic flow of phan-thien-tanner fluid has been studied by Vajravelu et al*.* in an asymmetric channel with porous medium^19^ and also Peristaltic flow and heat transfer of a Herschel-Bulkley fluid in an inclined non-uniform channel with wall properties is also done by Vajravelu et al*.*^20^.

The aim of the study is to check the mathematical behavior of non-Newtonian viscoelastic Eyring Powell Nanofluid having incorporated $\text{Al}_{2}{\text{O}}_{3}$ for Eyring Powell Parameter $M$, the porosity $V$ and the permeability parameter $W$. In our investigation, we have considered the transport bounded by two porous peristaltic plates in which the rate at which fluid is coming in the flow portion through one plate and the rate at which it is exiting the flow region through another plate is the same. This rate is called the porosity $V$ of the boundaries. Also, a free pumping case is considered. Physically, our aim is to study the blood flow in biological systems, improve and enhance the efficiency of energy extractions in underground reservoirs by checking the effect of various physical parameters like porosity, permeability and viscosity on the ‘velocity’ and ‘temperature’ of the fluid that is moving in human peristaltic membranes, underground water pipelines, extractions of minerals and any other physical systems.

## Mathematical formulation

**Figure 1:**
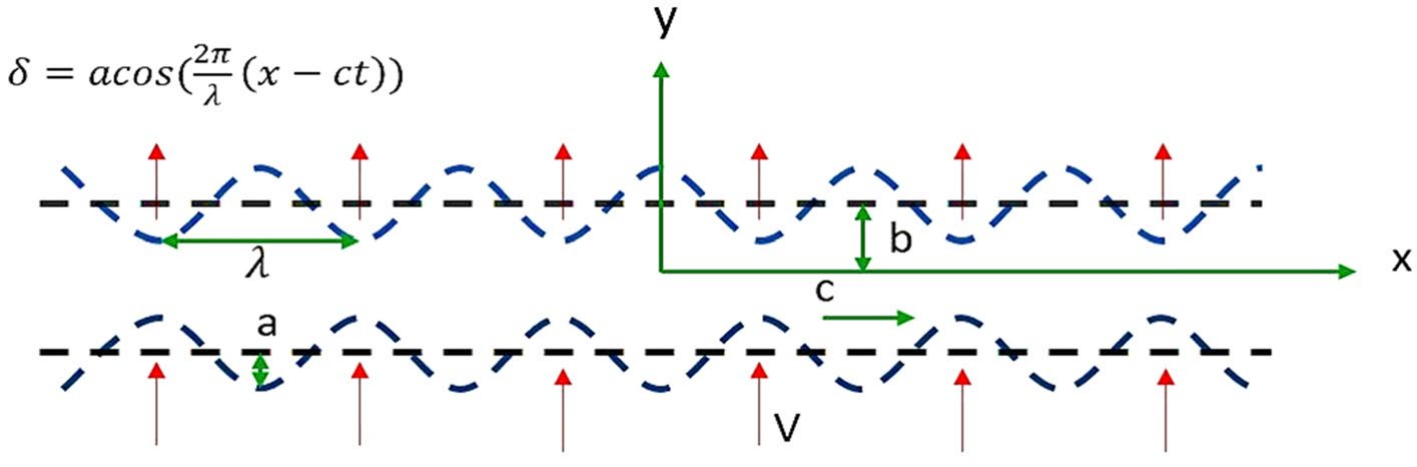
Flow geometry.

Consider an infinite 2d channel of thickness 2b, filled with an incompressible viscoelastic Eyring Powell Nanofluid containing $\text{Al}_{2}{\text{O}}_{3}$ as shown in Fig. 1. The walls of the channel are imposed to be small travelling sinusoidal waves having porosity and flexibility.

The equations which govern the incompressible flow is:

1 $$ div{\varvec{V}} = 0, $$

where $${\varvec{V}} = \left( {u, v} \right).$$

2 $$ \rho \frac{{d{\varvec{V}}}}{dt} = - \nabla p + div{\varvec{S}} + {\varvec{P}}, $$

The extra stress tensor **S** for Eyring Powell fluid is:

3 $$ \begin{aligned} & {\mathbf{S}} = \mu \nabla {\varvec{V}} + \frac{1}{\beta }sinh^{ - 1} \left( {\frac{1}{{c_{1} }}\nabla {\varvec{V}}} \right), \\ & {\mathbf{S}} = \mu \left( {1 + \frac{1}{{\beta c_{1} \mu }}} \right)\nabla^{2} { }{\varvec{V}},\\ \end{aligned} $$

with $$\left| {\frac{1}{{c_{1}^{3} }}\left( {\nabla {\varvec{V}}} \right)^{3} } \right| \ll < 1$$.

The fluid in a porous medium has the resistance:

4 $${\varvec{P}}=-\frac{{\mu }_{nf}}{W}\boldsymbol{ }\left(1+\frac{1}{\beta {c}_{1}{\mu }_{nf}}\right){\varvec{V}},$$

The governing two-dimensional momentum equations through a porous medium are:

5 $$\frac{\partial u}{\partial t}+u\frac{\partial u}{\partial x}+v\frac{\partial u}{\partial y}=-\frac{\partial p}{\partial x}+\frac{{\mu }_{nf}}{{\rho }_{nf}}\left(1+\frac{1}{\beta {c}_{1}{\mu }_{nf}}\right)\left(\frac{{\partial }^{2}u}{{\partial x}^{2}}+\frac{{\partial }^{2}u}{{\partial y}^{2}}\right)-\frac{{\mu }_{nf}}{{\rho }_{nf}W}\left(1+\frac{1}{\beta {c}_{1}\mu }\right)u,$$

6 $$\frac{\partial v}{\partial t}+u\frac{\partial v}{\partial x}+v\frac{\partial v}{\partial y}=-\frac{\partial p}{\partial y}+\frac{{\mu }_{nf}}{{\rho }_{nf}}\left(1+\frac{1}{\beta {c}_{1}{\mu }_{nf}}\right)\left(\frac{{\partial }^{2}v}{{\partial x}^{2}}+\frac{{\partial }^{2}v}{{\partial y}^{2}}\right)-\frac{{\mu }_{nf}}{{\rho }_{nf}W}\left(1+\frac{1}{\beta {c}_{1}\mu }\right)v,$$

7 $$\frac{\partial T}{\partial t}+u\frac{\partial T}{\partial x}+v\frac{\partial T}{\partial y}=\frac{k}{\rho {C}_{p}}\left(\frac{{\partial }^{2}T}{{\partial x}^{2}}+\frac{{\partial }^{2}T}{{\partial y}^{2}}\right),$$

The fluid in porous flexible walls is subjected to the boundary conditions having a vertical displacement of the lower wall $-\updelta$ and upper wall δ which is,

$$\updelta =\mathrm{acos}\left(\frac{2\pi }{\lambda }\left(x-ct\right)\right).$$

The boundary conditions are:

8 $$u(x,\pm b\pm\updelta ,\mathrm{t})=0,$$

9 $$v\left(y,\pm b\pm\updelta ,\mathrm{t}\right)=\mathrm{V}\pm \frac{\partial\updelta (\mathrm{x},\mathrm{t})}{\partial \mathrm{t}},$$

10 $$T\left(x,b+\updelta ,\mathrm{t}\right)=1,$$

11 $$T\left(x,-b-\updelta ,\mathrm{t}\right)=0.$$

where momentum equations and boundary conditions are taken from^6^ and energy equations from^18^.

For solving equations, we make stream function $\psi$ as $u = \frac{{\partial { }\psi { }}}{\partial y}$, $v = - \frac{{\partial { }\psi { }}}{\partial x}$ in Naiver stokes Eqs. (5), (6) and (7) and reduce these into

12 $$ \frac{{\partial \nabla^{2} \psi }}{\partial t} + \psi_{y} \nabla^{2} \psi_{x} - \psi_{x} \nabla^{2} \psi_{y} = \frac{{\mu_{nf} }}{{\rho_{nf} }}\left( {1 + \frac{1}{{\beta c_{1} \mu }}} \right)(\nabla^{4} \psi - \frac{1}{W}\nabla^{2} \psi ), $$

13 $$ \frac{\partial T}{{\partial t}} + \psi_{y} \frac{\partial T}{{\partial x}} - \psi_{x} \frac{\partial T}{{\partial y}} = \frac{{k_{nf} }}{{\rho_{nf} C_{{p_{nf} }} }}\left( {\frac{{\partial^{2} T}}{{\partial x^{2} }} + \frac{{\partial^{2} T}}{{\partial y^{2} }}} \right), $$

where

$$ \nabla^{2} = \left( {\frac{{\partial^{2} }}{{\partial x^{2} }} + \frac{{\partial^{2} }}{{\partial y^{2} }}} \right), $$

By putting the below non-dimensional parameters in Eqs. (8)–(13)

14 $$ \begin{aligned} & \overline{x} = \frac{x}{b}, \quad \overline{y} = \frac{y}{b}, \quad \overline{u} = \frac{u}{c}, \quad \overline{v} = \frac{v}{c}, \quad \overline{\psi } = \frac{\psi }{cb}, \quad \overline{V} = \frac{V}{c}, \quad {\overline{\delta }} = \frac{{\updelta }}{b}, \quad \overline{t} = \frac{ct}{b}, \\ & \overline{W} = \frac{W}{{b^{2} }}, \quad \overline{p} = \frac{p}{{\rho c^{2} }}, \quad A_{1} = \frac{{\mu_{nf} }}{{\mu_{f} }},\quad B_{1} = \frac{{\rho_{nf} }}{{\rho_{f} }}, \quad C_{1} = \frac{{k_{nf} }}{{k_{f} }},\quad D_{1} = \frac{{c_{{p_{nf} }} }}{{c_{{p_{f} }} }}, \quad {\text{R}} = \frac{cb\rho }{\mu }, \quad Pr = \frac{{\mu c_{p} }}{k} \\ & \alpha = \frac{2\pi b}{\lambda }, \quad \varepsilon = \frac{a}{b} ,\quad \theta = \frac{{T - T_{0} }}{{T_{1} - T_{0} }} \\ \end{aligned} $$

we get non-dimensional equations,

15 $$ \frac{{\partial \nabla^{2} \psi }}{\partial t} + \psi_{y} \nabla^{2} \psi_{x} - \psi_{x} \nabla^{2} \psi_{y} = \frac{1}{{RB_{1} }}\left( {A_{1} + M} \right)\nabla^{4} \psi - \frac{{A_{1} }}{{RWB_{1} }}\nabla^{2} \psi $$

16 $$ \frac{\partial \theta }{{\partial t}} + \psi_{y} \frac{\partial \theta }{{\partial x}} - \psi_{x} \frac{\partial \theta }{{\partial y}} = \frac{1}{Hr}\left( {\nabla^{2} \theta } \right) $$

17 $$ {\updelta } = \varepsilon {\text{cos}}\left( {\alpha \left( {x - t} \right)} \right) $$

18 $$ \psi_{y} \left( {x, \pm 1 \pm \updelta,{\text{t}}} \right) = 0 $$

19 $$ \psi_{x} \left( {x, \pm 1 \pm \updelta,{\text{t}}} \right) = - {\text{V}} \mp {{\varepsilon \alpha sin\alpha }}\left( {{\text{x}} - {\text{t}}} \right) $$

20 $$ \theta \left( {x,1,t} \right) = 1 $$

21 $$ \theta \left( {x, - 1,t} \right) = 0 $$

where $Hr = \frac{{C_{1} }}{{B_{1} D_{1} R Pr}}$ and according to ${\text{M}} = \frac{1}{{\beta c_{1} \mu }}$.Some characteristic formulae for Nanofluids are:

$$\begin{aligned} &\frac{{\mu }_{nf}}{{\mu }_{f}}=\frac{1}{{\left(1-{\varnothing }_{v}\right)}^{2.5}}\\ &\frac{{\rho }_{nf}}{{\rho }_{f}}=\left(1-{\varnothing }_{v}\right)+{\varnothing }_{v}\left(\frac{{\rho }_{p}}{{\rho }_{b}}\right)\\ &\frac{{k}_{nf}}{{k}_{f}}=\frac{{\mathrm{k}}_{P}+2{\mathrm{k}}_{b}-2{\varnothing }_{v}\left(\mathrm{kb}-{\mathrm{k}}_{P}\right)}{{\mathrm{k}}_{P}+2{\mathrm{k}}_{b}+{\varnothing }_{v}\left(\mathrm{kb}-{\mathrm{k}}_{P}\right)}\\& \frac{{c}_{{p}_{nf}}}{{c}_{{p}_{f}}}=\frac{{\varnothing }_{v} {\uprho }_{p} {\mathrm{c}}_{p}+\left(1-{\varnothing }_{v}\right){\uprho }_{b} {\mathrm{c}}_{b}}{{\varnothing }_{v} {\uprho }_{p}+(1-{\varnothing }_{v}){\uprho }_{b}} \end{aligned} $$

## Solution of the problem

To find the solution of Eq. (11), we apply a perturbation technique to $\psi$ and $\frac{\partial p}{\partial x}$ and perturbed boundary conditions as it is used by Nadeem et al.^10^. The term $({\frac{\partial p}{\partial x})}_{0}$ is an imposed steady pressure gradient. By equating the like powers ${\varepsilon }^{0}, {\varepsilon }^{1}$ and ${\varepsilon }^{2}$, we get sets of non-linear coupled 4th-order differential equations,

22 $$ \left( {\frac{{\partial \nabla^{2} \psi_{0} }}{\partial t}} \right) + \psi_{0y} \nabla^{2} \psi_{0x} - \psi_{0x} \nabla^{2} \psi_{0y} = \frac{1}{{RB_{1} }}\left( {A_{1} + M} \right)\nabla^{4} \psi_{0} - \frac{{A_{1} }}{{RWB_{1} }}\nabla^{2} \psi_{0} , $$

23 $$ \left( {\frac{{\partial \nabla^{2} \psi_{1} }}{\partial t}} \right) + \psi_{0y} \nabla^{2} \psi_{1x} + \psi_{1y} \nabla^{2} \psi_{0x} - \psi_{0x} \nabla^{2} \psi_{1y} - \psi_{1x} \nabla^{2} \psi_{0y} = \frac{1}{{RB_{1} }}\left( {A_{1} + M} \right)\nabla^{4} \psi_{1} - \frac{{A_{1} }}{{RWB_{1} }}\nabla^{2} \psi_{1} , $$

24 $$\frac{\partial {\nabla }^{2}{\psi }_{1}}{\partial t}+{\psi }_{0y}{\nabla }^{2}{\psi }_{2x}+{\psi }_{1y}{\nabla }^{2}{\psi }_{1x}+{\psi }_{2y}{\nabla }^{2}{\psi }_{0x}-{\psi }_{0x}{\nabla }^{2}{\psi }_{2y}-{\psi }_{1x}{\nabla }^{2}{\psi }_{1y}-{\psi }_{2x}{\nabla }^{2}{\psi }_{0y}=\frac{1}{{RB}_{1}}\left({A}_{1}+M\right){\nabla }^{4}{\psi }_{2}-\frac{{A}_{1}}{{RWB}_{1}}{\nabla }^{2}{\psi }_{2},$$

25 $$\frac{\partial {{\theta }_{T}}_{0}}{\partial t}+{\psi }_{0y}\frac{\partial {{\theta }_{T}}_{0}}{\partial x}-{\psi }_{0x}\frac{\partial {{\theta }_{T}}_{0}}{\partial y}=\frac{1}{Hr}\left({\nabla }^{2}{{\theta }_{T}}_{0}\right),$$

26 $$\frac{\partial {{\theta }_{T}}_{1}}{\partial t}+{\psi }_{0y}\frac{\partial {{\theta }_{T}}_{1}}{\partial x}+{\psi }_{1y}\frac{\partial {{\theta }_{T}}_{0}}{\partial x}-{\psi }_{0x}\frac{\partial {{\theta }_{T}}_{1}}{\partial y}-{\psi }_{1x}\frac{\partial {{\theta }_{T}}_{0}}{\partial y}=\frac{1}{Hr}\left({\nabla }^{2}{{\theta }_{T}}_{1}\right),$$

with boundary conditions

27 $${{\theta }_{T}}_{0}\left(x,-1,\mathrm{t}\right)=0,$$

28 $${{\theta }_{T}}_{0}\left(x,1,\mathrm{t}\right)=1,$$

29 $${{\theta }_{T}}_{1}\left(x, \pm 1,\mathrm{t}\right)\pm {{\theta }_{T}}_{0y}\left(x, \pm 1,\mathrm{t}\right)\mathrm{cos\alpha }\left(\mathrm{x}-\mathrm{t}\right)=0,$$

where boundary conditions of ${\psi }_{0}, {\psi }_{1}$ and ${\psi }_{2}$ are the same as used by Husseny and Shehawey in^6^.

- Solution of $${{{\psi}}}_{0}$$

Here we have used the same solution technique used by et al. Husseny^6^. As we know that the fluid enters the flow region through one plate at the same rate as it exits the other plate with velocity V so, the solution of ${\psi }_{0}$ and ${\theta }_{0}$ between two plates for steady-state flow is:

$${\psi }_{0}\left(x,y\right)=\frac{2kW{B}_{1}}{{A}_{1}\mathrm{sinh}\left({m}_{1}-{m}_{2}\right)}\left(ysinh\left({m}_{1}-{m}_{2}\right)+\frac{sinh\,{m}_{2}}{{m}_{1}}{e}^{{m}_{1}y}-\frac{sinh\,{m}_{1}}{{m}_{2}}{e}^{{m}_{2}y}\right)-Vx$$

where $2kW$ is the zeroth order of pressure gradient in which k is the pumping constant.

It should be noticed that the present problem is not an Eigenvalue, and the boundary conditions are not all homogenous as in all problems of hydrodynamic stabilities. So, to solving ${\psi }_{1}$ and ${\psi }_{2}$ we restrict our study to free pumping case. Thus, in our case k = 0 and we can obtain a closed-form simple analytic solution.

30 $${\psi }_{0}\left(x\right)=-Vx$$

with

31 $$\begin{aligned} &{m}_{1}=\frac{\frac{RV{B}_{1}}{({A}_{1}+M)}+\sqrt{{\left(\frac{RV{B}_{1}}{{A}_{1}+M}\right)}^{2}+\frac{4{A}_{1}}{{(A}_{1}+M)W}}}{2}, {m}_{2}=\frac{\frac{RV{B}_{1}}{({A}_{1}+M)}-\sqrt{{\left(\frac{RV{B}_{1}}{{A}_{1}+M}\right)}^{2}+\frac{4{A}_{1}}{{(A}_{1}+M)W}}}{2}\\ & {\theta }_{0}\left(y\right)=\frac{1}{1-{e}^{2HrV}}\left(1-{e}^{HrV\left(y+1\right)}\right). \end{aligned}$$

- Solutions of ${{{\psi}}}_{1}, {{\psi}}_{2}$ and ${{{\theta}}}_{1}$

The following type of solution according to^6,18^ is satisfied by ${\psi }_{1}, {\psi }_{2},$ and ${\theta }_{1}$ with its corresponding boundary conditions:

32 $${\psi }_{1}=\frac{1}{2}\left({\varphi }_{1}\left(y\right){e}^{i\alpha \left(x-t\right)}+{{\varphi }^{*}}_{1}\left(y\right){e}^{-i\alpha \left(x-t\right)}\right),$$

33 $${\psi }_{2}\left(x,y\right)=\frac{1}{2}\left({\varphi }_{20}\left(y\right)+{\varphi }_{22}\left(y\right){e}^{2i\alpha \left(x-t\right)}+{{\varphi }^{*}}_{22}\left(y\right){e}^{-2i\alpha \left(x-t\right)}\right),$$

34 $${\theta }_{1}\left(x,y\right)={h}_{1}\left(y\right){e}^{i\alpha \left(x-t\right)},$$

where * represents the complex conjugate. Now, substituting Eqs. (31)–(34) in Eqs. (19)–(24) we get the following ordinary differential equation in terms of $\varphi$ and $h$.

35 $$\left(\frac{{d}^{2}}{{dy}^{2}}-{\alpha }^{2}+\frac{i\alpha R{B}_{1}}{({A}_{1}+M)}-\frac{{A}_{1}}{({A}_{1}+M)W}\right)\left(\frac{{d}^{2}}{{dy}^{2}}-{\alpha }^{2}\right)\left({{\varphi }_{1}}^{{\prime}}\right)-\frac{RV{B}_{1}}{\left({A}_{1}+M\right)}\left(\frac{{d}^{2}}{{dy}^{2}}-{\alpha }^{2}\right)\left({\varphi }_{1}\right) =0,$$

36 $${{\varphi }_{20}}^{\prime\prime\prime\prime}-\frac{RV{B}_{1}}{\left({A}_{1}+M\right)}{{\varphi }_{20}}^{\prime\prime\prime}-\frac{{A}_{1}}{\left({A}_{1}+M\right)W}{{\varphi }_{20}}^{\prime\prime}=\frac{i\alpha R{B}_{1}}{2\left({A}_{1}+M\right)}{\left({{\varphi }^{*}}_{1}{{\varphi }_{1}}^{\prime\prime}-{\varphi }_{1}{{\varphi }_{1}}^{{*}^{\prime\prime}}\right)}^{{\prime}},$$

37 $$\frac{{d}^{2}{h}_{1}}{{dy}^{2}}-HrV\frac{d{h}_{1}}{dx}-Hr\left({\alpha }^{2}-i\alpha \right){h}_{1}=-\frac{1}{2}i\alpha Hr{\varphi }_{1},$$

with boundary conditions

38 $${{\varphi }_{1}}^{{\prime}}\left(\pm 1\right)=0,$$

39 $${\varphi }_{1}\left(\pm 1\right)=\pm 1,$$

40 $${{\varphi }_{20}}^{{\prime}}\left(\pm 1\right)=\mp \frac{1}{2}\left({{\varphi }_{1}}^{\prime\prime}\left(\pm 1\right)+{{\varphi }_{1}{*}^{{\prime}}}^{{\prime}}\left(\pm 1\right)\right),$$

41 $${h}_{1}\left( \pm 1\right)=-{\theta }_{0y}\left( \pm 1\right),$$

where $${\theta }_{0y}=\frac{{e}^{HVy}}{{e}^{2HV}-1}$$.

By solving (35), (36) and (37) we get,

42 $${\varphi }_{1}={D}_{1}cosh\alpha y+{D}_{2}sinh\alpha y{+D}_{3}{e}^{{\theta }_{1}y}{+D}_{4}{e}^{{\theta }_{2}y} .$$

Since we simply want to get the mean flow, so we only need to solve ${\psi }_{2}$ for ${{\varphi }_{20}}^{{\prime}}$ The solution to Eq. (36) is thus,

43 $$\begin{aligned} &{{\varphi }_{20}}^{{\prime}}=H\left(y\right)+\frac{H\left(1\right)\left({e}^{-{m}_{1}+{m}_{2}y}-{e}^{-{m}_{2}+{m}_{1}y}\right)+H\left(-1\right)\left({e}^{{m}_{2}+{m}_{1}y}-{e}^{{m}_{1}+{m}_{2}y}\right)}{2\,\mathrm{sinh}({m}_{1}-{m}_{2})}\\ &\quad +{E}_{1}\left[2W\frac{({A}_{1}+M)}{{B}_{1}} \left(\frac{{e}^{{m}_{2}y}\mathrm{sinh}\,{m}_{1-{e}^{{m}_{1}y}\mathrm{sinh},{m}_{2}}}{\mathrm{sinh}({m}_{1}-{m}_{2})}-1\right)\right]\\ &\quad+\frac{{E}_{2}\left[{e}^{-{m}_{2}+{m}_{1}y}-{e}^{-{m}_{1}+{m}_{2}y}\right]+{E}_{3}[{e}^{{m}_{1}+{m}_{2}y}-{e}^{{m}_{2}+{m}_{1}y}]}{2\,\mathrm{sinh}({m}_{1}-{m}_{2})}, \end{aligned}$$

44 $$ \begin{aligned} {h}_{1}\left(y\right)&={F}_{1}{e}^{{w}_{1}}+{F}_{2}{e}^{{w}_{2}}-\frac{1}{2}i\alpha {Hr}^{2}VJ[\frac{{D}_{1}}{2{K}_{1}}{e}^{\left(HrV+\alpha \right)y}+\frac{{D}_{1}}{2{K}_{2}}{e}^{\left(HrV-\alpha \right)y}+\frac{{D}_{2}}{2{K}_{1}}{e}^{\left(HrV+\alpha \right)y}\\ &\quad -\frac{{D}_{2}}{2{K}_{2}}{e}^{\left(HrV-\alpha \right)y}+\frac{{D}_{3}}{{K}_{3}}{e}^{\left(HrV+{\theta }_{1}\right)y}+\frac{{D}_{4}}{{K}_{4}}{e}^{\left(HrV+{\theta }_{2}\right)y}. \end{aligned}$$

By using (38)–(41) in (42)–(44) we get

$$\begin{aligned} &{\theta }_{1}=\frac{\frac{RV{B}_{1}}{({A}_{1}+M)}+\sqrt{{\left(\frac{RV{B}_{1}}{({A}_{1}+M)}\right)}^{2}+4s}}{2}, \\  &{\theta }_{2}=\frac{\frac{RV{B}_{1}}{({A}_{1}+M)}-\sqrt{{\left(\frac{RV{B}_{1}}{({A}_{1}+M)}\right)}^{2}+4s}}{2},\\ & s={\alpha }^{2}-\frac{i\alpha R{B}_{1}}{\left({A}_{1}+M\right)}+\frac{{A}_{1}}{\left({A}_{1}+M\right)W}, \\ & {D}_{4}=\frac{-(\alpha {s}_{3}cosh\alpha )}{{s}_{3}{s}_{2}-{s}_{1}{s}_{4}}, \\& {D}_{3}=\frac{(\alpha {s}_{4}cosh\alpha )}{{s}_{3}{s}_{2}-{s}_{1}{s}_{4}},\\ &{D}_{1}=\frac{1-{D}_{3}sinh{\theta }_{1}-{D}_{4}sinh{\theta }_{2}}{sinh\alpha },\\& {D}_{2}=\frac{-({D}_{3}cosh{\theta }_{1}+{D}_{4}cosh{\theta }_{2}}{cosh\alpha },\\ &{s}_{1}={\theta }_{1}sinh\alpha\,cosh{\theta }_{1}-\alpha sinh{\theta }_{1}\,cosh\alpha ,\\& {s}_{2}={\uptheta }_{2}sinh\alpha\,cosh{\theta }_{2}-\alpha sinh{\theta }_{2}\,cosh\alpha ,\\& {s}_{3}={\theta }_{1}cosh\,\alpha sinh{\theta }_{1}-\alpha\,cosh{\theta }_{1} \,sinh\alpha ,\\& {s}_{4}={\theta }_{2}cosh\alpha\,sinh{\theta }_{2}-\alpha\,cosh{\theta }_{2}\,sinh\alpha,\\ &H\left(y\right)=\frac{i\alpha R{B}_{1}}{2\left({A}_{1}+M\right)}\{[\frac{{D}_{3}{d}_{1}\left({{\theta }_{1}}^{2}-{\alpha }^{2}\right){e}^{{\theta }_{1}y}}{{{d}_{1}}^{2}-{{d}_{2}}^{2}}+\frac{{D}_{4}{d}_{3}\left({{\theta }_{2}}^{2}-{\alpha }^{2}\right){e}^{{\theta }_{2}y}}{{{d}_{3}}^{2}-{{d}_{4}}^{2}}]({{D}_{1}}^{*}\mathrm{cosh}\alpha y+{{D}_{2}}^{*}\mathrm{sinh}\alpha y)\\ &\quad\quad-[\frac{{D}_{3}{d}_{2}\left({{\theta }_{1}}^{2}-{\alpha }^{2}\right){e}^{{\theta }_{1}y}}{{{d}_{1}}^{2}-{{d}_{2}}^{2}}+\frac{{D}_{4}{d}_{4}\left({{\theta }_{2}}^{2}-{\alpha }^{2}\right){e}^{{\theta }_{2}y}}{{{d}_{3}}^{2}-{{d}_{4}}^{2}}]({{D}_{1}}^{*}\mathrm{sinh}\alpha y+{{D}_{2}}^{*}\mathrm{cosh}\alpha y) \\ &\quad\quad+[\frac{{{D}_{3}}^{*}{{d}_{1}}^{*}\left({\alpha }^{2}-{{{\theta }_{1}}^{*}}^{2}\right){e}^{{{\theta }_{1}}^{*}y}}{{{d}_{1}}^{*2}-{{d}_{2}}^{*2}}+\frac{{{D}_{4}}^{*}{{d}_{3}}^{*}\left({\alpha }^{2}-{{{\theta }_{2}}^{*}}^{2}\right){e}^{{{\theta }_{2}}^{*}y}}{{{d}_{3}}^{*2}-{{d}_{4}}^{*2}}]({D}_{1}\mathrm{cosh}\alpha y+{D}_{2}\mathrm{sinh}\alpha y)\\ &\quad\quad-[ \frac{{{D}_{3}}^{*}{{d}_{2}}^{*}\left({\alpha }^{2}-{{{\theta }_{1}}^{*}}^{2}\right){e}^{{{\theta }_{1}}^{*}y}}{{{d}_{1}}^{*2}-{{d}_{2}}^{*2}}+\frac{{{D}_{4}}^{*}{{d}_{4}}^{*}\left({\alpha }^{2}-{{{\theta }_{2}}^{*}}^{2}\right){e}^{{{\theta }_{2}}^{*}y}}{{{d}_{3}}^{*2}-{{d}_{4}}^{*2}}]({D}_{1}\mathrm{sinh}\alpha y+{D}_{2}\mathrm{cosh}\alpha y)\\  &\quad\quad+\frac{{{D}_{3}}^{*}{D}_{3}\left({{\theta }_{1}}^{2}-{{{\theta }_{1}}^{*}}^{2}\right){e}^{\left({\theta }_{1}+{{\theta }_{1}}^{*}\right)y}}{{\left({\theta }_{1}+{{\theta }_{1}}^{*}\right)}^{2}- \frac{RV{B}_{1}}{{A}_{1}\left({B}_{1}+M\right)}\left({\theta }_{1}+{{\theta }_{1}}^{*}\right)-\frac{{B}_{1}}{{A}_{1}\left({B}_{1}+M\right)W}}+\frac{{{D}_{4}}^{*}{D}_{3}\left({{\theta }_{1}}^{2}-{{{\theta }_{2}}^{*}}^{2}\right){e}^{\left({\theta }_{1}+{{\theta }_{2}}^{*}\right)y}}{{\left({\theta }_{1}+{{\theta }_{2}}^{*}\right)}^{2}- \frac{RV{B}_{1}}{{A}_{1}\left({B}_{1}+M\right)}\left({\theta }_{1}+{{\theta }_{2}}^{*}\right)-\frac{{B}_{1}}{{A}_{1}\left({B}_{1}+M\right)W}}\\&\quad\quad +\frac{{{D}_{3}}^{*}{D}_{4}\left({{\theta }_{2}}^{2}-{{{\theta }_{1}}^{*}}^{2}\right){e}^{\left({\theta }_{2}+{{\theta }_{1}}^{*}\right)y}}{{\left({\theta }_{2}+{{\theta }_{1}}^{*}\right)}^{2}- \frac{RV{B}_{1}}{{A}_{1}\left({B}_{1}+M\right)}\left({\theta }_{2}+{{\theta }_{1}}^{*}\right)-\frac{{B}_{1}}{{A}_{1}\left({B}_{1}+M\right)W}}+\frac{{{D}_{4}}^{*}{D}_{4}\left({{\theta }_{2}}^{2}-{{{\theta }_{2}}^{*}}^{2}\right){e}^{\left({\theta }_{2}+{{\theta }_{2}}^{*}\right)y}}{{\left({\theta }_{2}+{{\theta }_{2}}^{*}\right)}^{2}- \frac{RV{B}_{1}}{{A}_{1}\left({B}_{1}+M\right)}\left({\theta }_{2}+{{\theta }_{2}}^{*}\right)-\frac{{B}_{1}}{{A}_{1}\left({B}_{1}+M\right)W}}, \end{aligned}$$

$$\begin{aligned} &{d}_{1}=\left({\alpha }^{2}+{{\theta }_{1}}^{2}-\frac{RV{B}_{1}}{\left({A}_{1}+M\right)}{\theta }_{1}-\frac{{A}_{1}}{\left({A}_{1}+M\right)W}\right),\\& {d}_{2}=\alpha \left(2{\theta }_{1}-\frac{RV{B}_{1}}{\left({A}_{1}+M\right)}\right), {d}_{3}=\left({\alpha }^{2}+{{\theta }_{2}}^{2}-\frac{RV{B}_{1}}{\left({A}_{1}+M\right)}{\theta }_{2}-\frac{{A}_{1}}{\left({A}_{1}+M\right)W}\right),\\& {d}_{4}=\alpha \left(2{\theta }_{2}-\frac{RV{B}_{1}}{\left({A}_{1}+M\right)}\right),\\& {E}_{2}={{\varphi }_{20}}^{{\prime}}\left(+1\right)=-\frac{1}{2}\{{\alpha }^{2}\left[\mathit{cos}h\alpha ({D}_{1}+{{D}_{1}}^{*}\right)+\mathit{sin}h\alpha ({D}_{2}+{{D}_{2}}^{*})]+ {{\theta }_{1}}^{2}{D}_{3}{e}^{{\theta }_{1}}+ {{\theta }_{2}}^{2}{D}_{4}{e}^{{\theta }_{2}}\\ &\quad+{{{\theta }_{1}}^{*}}^{2}{{D}_{3}}^{*}{e}^{{{\theta }_{1}}^{*}}+{{{\theta }_{2}}^{*}}^{2}{{D}_{4}}^{*}{e}^{{{\theta }_{2}}^{*}},\\& {E}_{3}={{\varphi }_{20}}^{{\prime}}=-\frac{1}{2}\{{\alpha }^{2}\left[\mathit{cos}h\alpha ({D}_{1}+{{D}_{1}}^{*}\right)-\mathit{sin}h\alpha ({D}_{2}+{{D}_{2}}^{*})]+ {{\theta }_{1}}^{2}{D}_{3}{e}^{{-\theta }_{1}}+ {{\theta }_{2}}^{2}{D}_{4}{e}^{-{\theta }_{2}}\\&\quad +{{{\theta }_{1}}^{*}}^{2}{{D}_{3}}^{*}{e}^{{{-\theta }_{1}}^{*}}+{{{\theta }_{2}}^{*}}^{2}{{D}_{4}}^{*}{e}^{{{-\theta }_{2}}^{*}},\\& {w}_{1}=\frac{HrV+\sqrt{{\left(HrV\right)}^{2}+4Hr\left(\alpha -i{\alpha }^{2}\right)}}{2},\\& {w}_{2}=\frac{HrV+\sqrt{{\left(HrV\right)}^{2}+4Hr(\alpha -i{\alpha }^{2})}}{2},\\& J=\frac{{e}^{HrV}}{{e}^{2HrV}-1} ,\\& Hr= \frac{{B}_{1}}{{C}_{1}{D}_{1}R Pr},\end{aligned}   $$

$$\begin{aligned} {K}_{1}&={\left(Hr\, V+\alpha \right)}^{2}-\left({\left(Hr\, V\right)}^{2}+\alpha Hr \,V\right)-\left({\alpha }^{2}-Hr\, i\, \alpha \right),\\ {K}_{2}&={\left(Hr \,V-\alpha \right)}^{2}-\left({\left(Hr\, V\right)}^{2}-\alpha Hr\, V\right)-\left({\alpha }^{2}-Hr\, i\, \alpha \right),\\  {K}_{3}&={({\theta }_{1}+Hr\, V)}^{2}-Hr \,V\left({\theta }_{1}+Hr \,V\right)-\left({\alpha }^{2}-Hr\, i\, \alpha \right),\\  {K}_{4}&={\left({\theta }_{2}+Hr\, V\right)}^{2}-Hr \,V\left({\theta }_{2}+Hr \,V\right)-\left({\alpha }^{2}-Hr\, i\, \alpha \right),\\  {F}_{3}&=\frac{{e}^{{\mathrm{w}}_{1}+{\mathrm{w}}_{2}}}{4{\mathrm{K}}_{1}{\mathrm{K}}_{2}{\mathrm{K}}_{3}{\mathrm{K}}_{4}\left(-{e}^{2{\mathrm{w}}_{1}}+{e}^{2{\mathrm{w}}_{2}}\right)\left(-1+{e}^{2Hr\, V}\right)}({e}^{{\mathrm{w}}_{2}} Hr\, V(4 {\mathrm{K}}_{1} {\mathrm{K}}_{2} {\mathrm{K}}_{3}{\mathrm{K}}_{4}\\ &\quad +i{e}^{-Hr\, V-{\theta }_{1}-{\theta }_{2}-\alpha }\left(-1+{e}^{2Hr\, V}\right)\left(2{\mathrm{K}}_{1}{\mathrm{K}}_{2}{e}^{\alpha }\left({\mathrm{D}}_{4}{\mathrm{K}}_{3}{e}^{{\theta }_{1}}+{\mathrm{D}}_{3}{\mathrm{K}}_{4}{e}^{{\theta }_{2}}\right) \right.\\&\quad  \left.+{\mathrm{D}}_{2}{\mathrm{K}}_{3}{\mathrm{K}}_{4}{e}^{{\theta }_{1}+{\theta }_{2}}\left({\mathrm{K}}_{2}-{\mathrm{K}}_{1}{e}^{2\alpha }\right)+{\mathrm{D}}_{1}{\mathrm{K}}_{3}{\mathrm{K}}_{4}{e}^{{\theta }_{1}+{\theta }_{2}}\left({\mathrm{K}}_{2}+{\mathrm{K}}_{1}{e}^{2\alpha }\right)\right)Hr\, J\alpha )\\&\quad+{e}^{-{\mathrm{w}}_{2}+HV}Hr\, V(4{\mathrm{K}}_{1}{\mathrm{K}}_{2}{\mathrm{K}}_{3}{\mathrm{K}}_{4}{e}^{Hr\, V} -i{e}^{-\alpha }\left(-1+{e}^{2Hr\, V}\right)\left(2{\mathrm{K}}_{1}{\mathrm{K}}_{2}{e}^{\alpha }\left({\mathrm{D}}_{3}{\mathrm{K}}_{4}{e}^{{\theta }_{1}}+{\mathrm{D}}_{4}{\mathrm{K}}_{3}{e}^{{\theta }_{2}}\right)\right. \\&\quad \left.-{\mathrm{D}}_{2}{\mathrm{K}}_{3}{\mathrm{K}}_{4}\left({\mathrm{K}}_{1}-{\mathrm{K}}_{2}{e}^{2\alpha }\right)+{\mathrm{D}}_{1}{\mathrm{K}}_{3}{\mathrm{K}}_{4}\left({\mathrm{K}}_{1}+{\mathrm{K}}_{2}{e}^{2\alpha }\right)\right)Hr\, J\,\alpha ))\\  {F}_{4}&= \frac{i{e}^{{\mathrm{w}}_{1}-Hr\, V-{\theta }_{1}-{\theta }_{2}-\alpha }Hr\, V}{4{\mathrm{K}}_{1}{\mathrm{K}}_{2}{\mathrm{K}}_{3}{\mathrm{K}}_{4}\left(-{e}^{2{\mathrm{w}}_{1}}+{e}^{2{\mathrm{w}}_{2}}\right)\left(-1+{e}^{2Hr\, V}\right)}(\left({D}_{1}-{\mathrm{D}}_{2}\right){\mathrm{K}}_{1}{\mathrm{K}}_{3}{\mathrm{K}}_{4}{e}^{{\theta }_{1}+{\theta }_{2}}\left(-1+{e}^{2Hr\, V}\right)\\&\quad\left({e}^{2Hr\, V}-{e}^{2\left({\mathrm{w}}_{1}+\alpha \right)}\right)Hr\,J\alpha +\left({D}_{1}  + {\mathrm{D}}_{2}\right){\mathrm{K}}_{2}{\mathrm{K}}_{3}{\mathrm{K}}_{4}{e}^{{\theta }_{1}+{\theta }_{2}}\left(-1+{e}^{2Hr\, V}\right)\left(-{e}^{2{\mathrm{w}}_{1}}+{e}^{2\left(Hr\, V+\alpha \right)}\right)Hr\,J\alpha \\&\quad +2{\mathrm{K}}_{1}{\mathrm{K}}_{2}{e}^{\alpha }({\mathrm{D}}_{3}{K}_{4}{e}^{{\theta }_{2}}\left(-1+{e}^{2Hr\, V}\right)\left(-{e}^{2{\mathrm{w}}_{1}1}+{e}^{2\left(Hr\, V+{\theta }_{1}\right)}\right)Hr\,J\alpha +{\mathrm{K}}_{3}{e}^{{\theta }_{1}}(2i{\mathrm{K}}_{4}{e}^{Hr\, V+{\theta }_{2}}\left({e}^{2{\mathrm{w}}_{1}}+{e}^{2Hr\, V}\right)\\&\quad+{\mathrm{D}}_{4}\left(-1+{e}^{2Hr\, V}\right)\left(-{e}^{2{\mathrm{w}}_{1}}+{e}^{2\left(Hr\, V+{\theta }_{2}\right)}\right)Hr\,J\alpha ))), \end{aligned}$$

By using the values of the above constants, we see that there is still an arbitrary constant ${E}_{1}$ in solution which is related to the time average second perturbed term of the pressure gradient. The solution found by using the perturbation equations of $\psi$, $\left(\frac{\partial p}{\partial x}\right)$ and (24) in Eq. (5), we get

$$\frac{{E}_{1}({A}_{1}+M)}{R{B}_{1}}={(\frac{\partial \overline{p}}{\partial x })}_{2}$$

So, the solution of velocity becomes:

$$u={{\psi }_{0}}_{y}+\in {{\psi }_{1}}_{y}+{\epsilon }^{2}{{\psi }_{2}}_{y},$$

$$u=\in {{\varphi }_{1}}^{{\prime}}{e}^{i\alpha \left(x-t\right)}+\frac{{\epsilon }^{2}}{2}{{\varphi }_{20}}^{{\prime}},$$

45 $$u=\in ({D}_{1}\alpha sinh\,\alpha y+{D}_{2}\alpha\,cosh\,\alpha y{+D}_{3}{{\theta }_{1}e}^{{\theta }_{1}y}{+D}_{4}{\theta }_{2}{e}^{{\theta }_{2}y}){e}^{i\alpha \left(x-t\right)}+\frac{{\epsilon }^{2}}{2}{{\varphi }_{20}}^{{\prime}}\{H\left(y\right)+\frac{H\left(1\right)\left({e}^{-{m}_{1}+{m}_{2}y}-{e}^{-{m}_{2}+{m}_{1}y}\right)+H\left(-1\right)\left({e}^{{m}_{2}+{m}_{1}y}-{e}^{{m}_{1}+{m}_{2}y}\right)}{2\,\mathrm{sinh}({m}_{1}-{m}_{2})}+2WR{\left(\frac{\partial p}{\partial x}\right)}_{2}\left(\frac{{e}^{{m}_{2}y}\mathrm{sinh}{m}_{1-{e}^{{m}_{1}y}\mathrm{sinh}{m}_{2}}}{\mathrm{sinh}({m}_{1}-{m}_{2})}-1\right)+\frac{{E}_{2}\left[{e}^{-{m}_{2}+{m}_{1}y}-{e}^{-{m}_{1}+{m}_{2}y}\right]+{E}_{3}\left[{e}^{{m}_{1}+{m}_{2}y}-{e}^{{m}_{2}+{m}_{1}y}\right]}{2\,\mathrm{sinh}({m}_{1}-{m}_{2})},$$

46 $$ \begin{aligned} & T = \theta_{0} + \in \theta_{1} ,\\ & T\left( {x,y} \right) = \frac{1}{{1 - e^{2HrV} }}\left( {1 - e^{{HrV\left( {y + 1} \right)}} } \right) + \varepsilon h_{1} \left( y \right)e^{{i\alpha \left( {x - t} \right)}} . \end{aligned} $$

## Graphical evaluations and analysis

Numerical computation is done to comprehend the impact of the channel’s porosity and the medium’s permeability parameter on the mean-time average velocity. A detailed examination of Eq. (46) reveals that the mean velocity of the fluid has major constants $$ E_{2} ,E_{3}$$ and the term $$2RW\left( {\frac{\partial p}{{\partial x}}} \right)_{2} \left( {\frac{{e^{{m_{2} y}} \sinh m_{{1 - e^{{m_{1} y}} \sinh m_{2} }} }}{{\sinh (m_{1} - m_{2} )}} - 1} \right)$$.

The Eqs. (45) and (46) determines the direction of the peristaltic flow of the transport, controls the velocity of the channel and determines the temperature changes among channel and boundary.

The value of $\varphi_{20}^{{\prime}}$ is responsible for $E_{2}$ and $E_{3}$ which are the result of the no-slip condition of the velocity at the boundary, are due to the value of $\varphi_{20}^{{\prime}}$ and link to the mean velocity at cross-section as:

$$ u\left( { + 1} \right) = \frac{{\epsilon^{2} }}{2}E_{2 = } \frac{{\epsilon^{2} }}{2}\varphi_{20}^{{\prime}} \left( { + 1} \right),\quad u\left( { - 1} \right) = \frac{{\epsilon^{2} }}{2}E_{3} = \frac{{\epsilon^{2} }}{2}\varphi_{20}^{{\prime}} \left( { - 1} \right), $$

which indicates that no slip is applied to the peristaltic boundary rather than the mean position of the wall. In our inquiry $\varphi_{20}^{{\prime}} \left( { + 1} \right) \ne \varphi_{20}^{{\prime}} \left( { - 1} \right)$ which means that the fluid motion is non-symmetric. This behavior is not like most investigations, but similar behavior is observed in the inquiry of Shehawey^8^.

**Figure 2:**
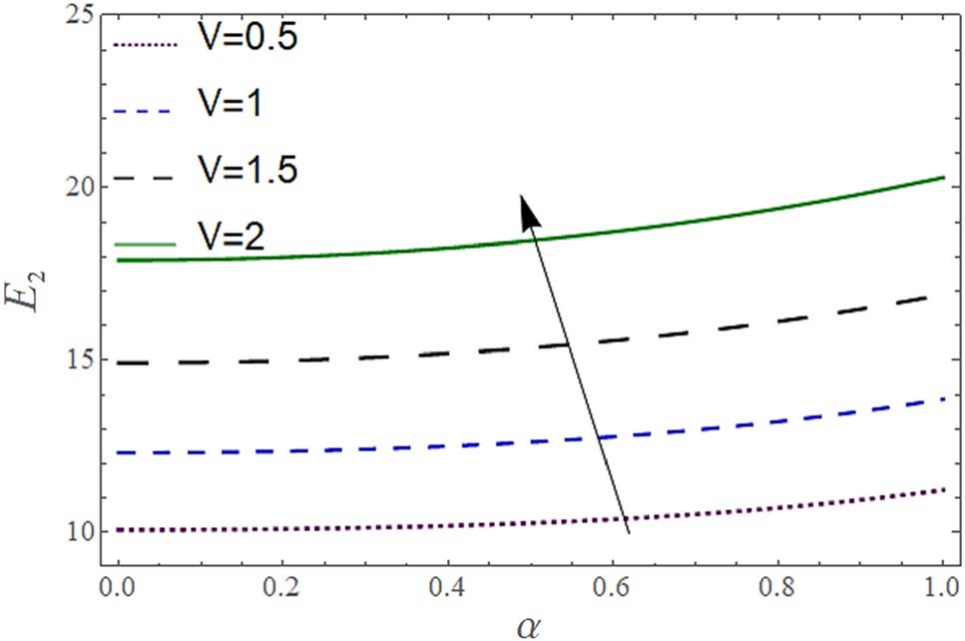
Behavior of $E_{2} { }$ with “*α*” for distinct values of V for $W = 0.002, M = 1, {\o} = 0.1, \rho_{p} = 3970, \rho_{b} = 997.1, R = 10$.

**Figure 3:**
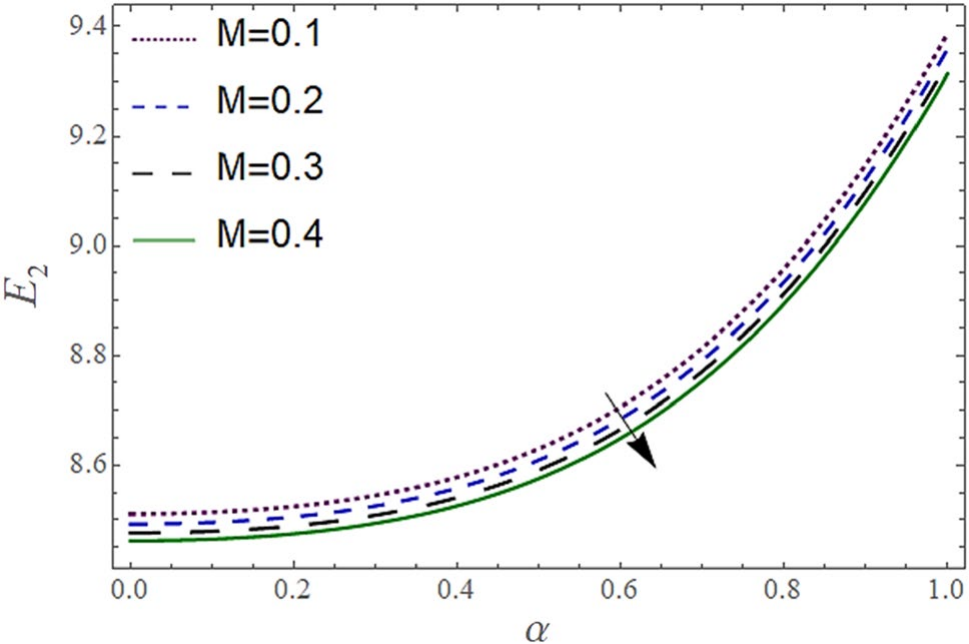
Behavior of $E_{2} { }$ with “*α*” for distinct values of M for $W = 0.002, V = 0.05, {\o} = 0.1, \rho_{p} = 3970, \rho_{b} = 997.1, R = 10$.

**Figure 4:**
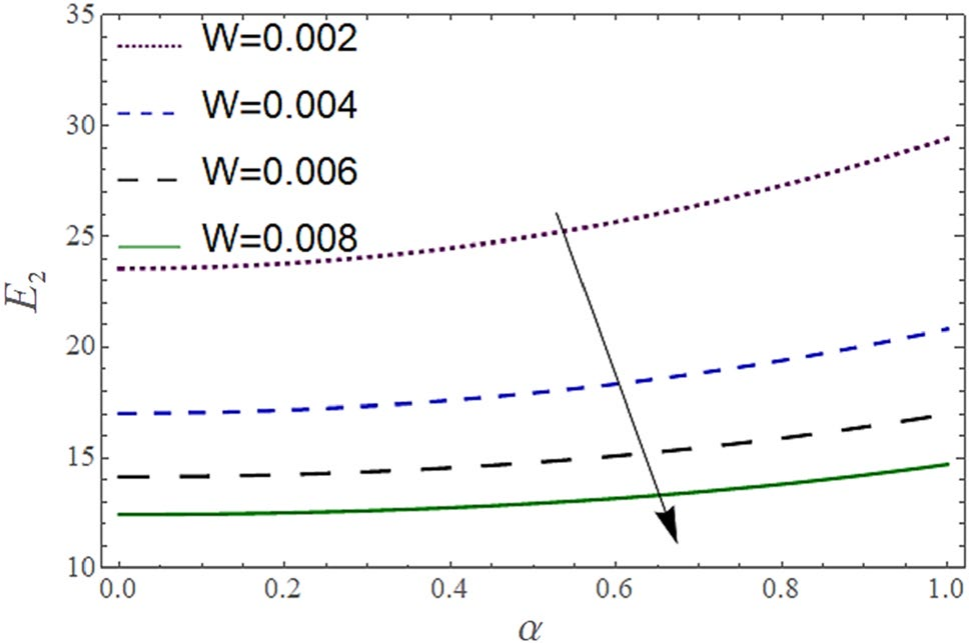
Behavior of $E_{2} { }$ with “*α*” for distinct values of W for $M = 1, V = 0.05, {\o} = 0.1, \rho_{p} = 3970, \rho_{b} = 997.1, R = 10$.

**Figure 5:**
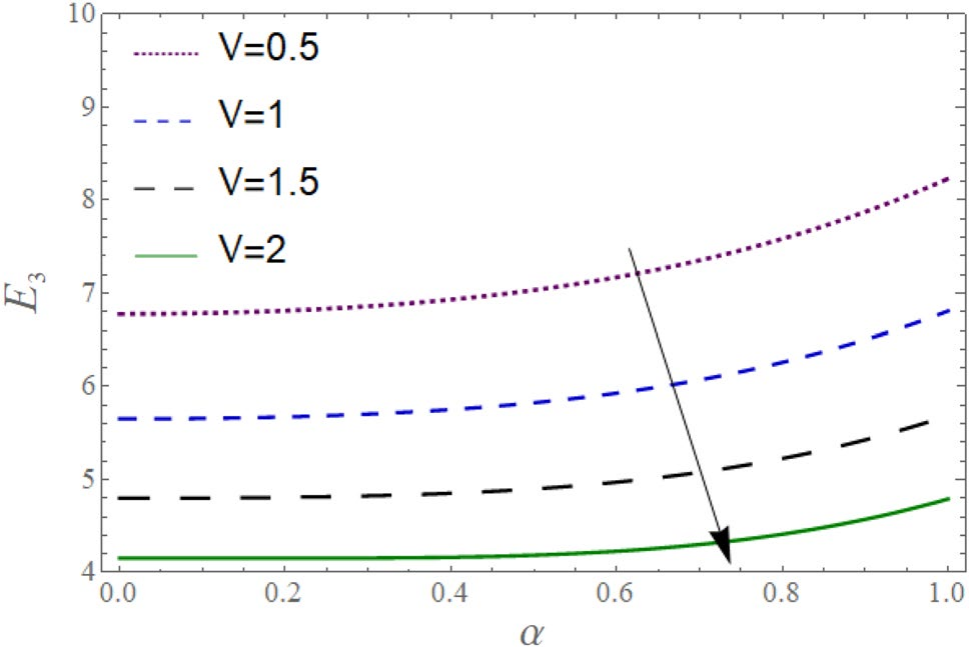
Behavior of $E_{3} { }$ with “*α*” for distinct values of V for $M = 1, W = 0.002, {\o} = 0.1, \rho_{p} = 3970, \rho_{b} = 997.1, R = 10$.

**Figure 6:**
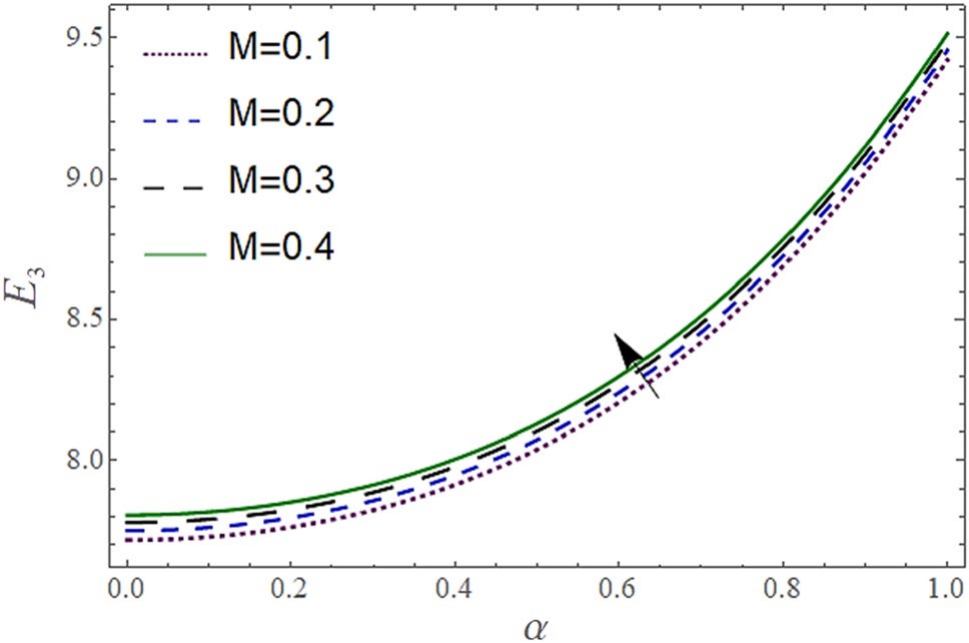
Behavior of $E_{3} { }$ with “*α*” for distinct values of M for $V = 0.05, W = 0.002, {\o} = 0.1, \rho_{p} = 3970, \rho_{b} = 997.1, R = 10$.

**Figure 7:**
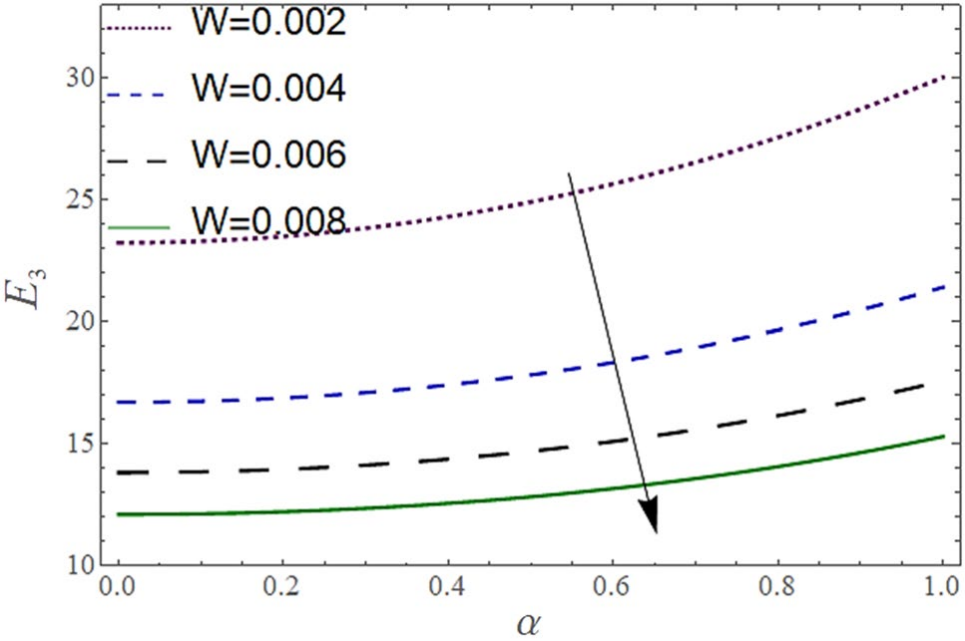
Behavior of $E_{2} { }$ with “*α*” for distinct values of V for $M = 1, W = 0.002, {\o} = 0.1, \rho_{p} = 3970, \rho_{b} = 997.1, R = 10$.

**Figure 8:**
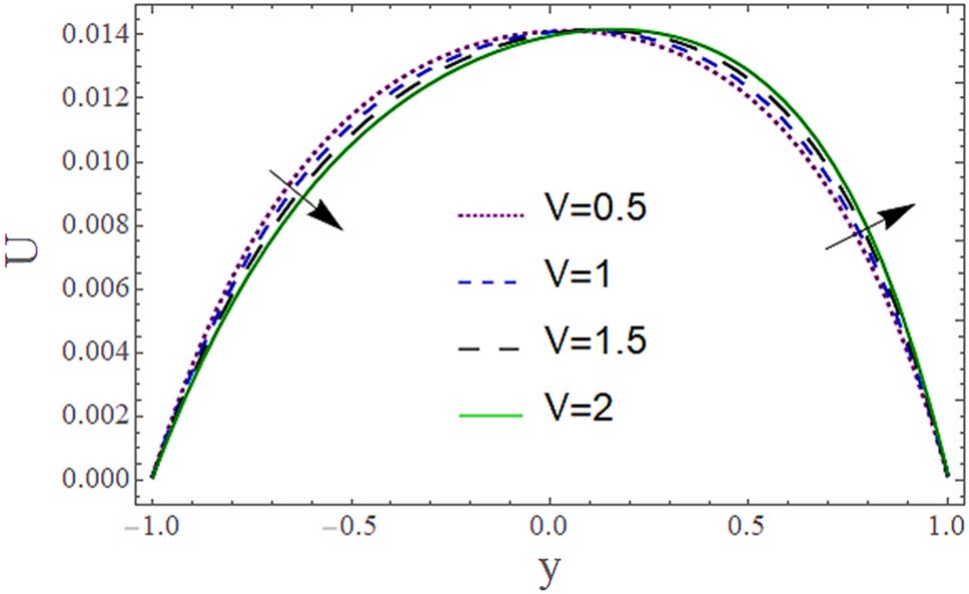
Behavior of $U{ }$ with “*y*” for distinct values of V for $W = 0.002, M = 1, {\o} = 0.1, \rho_{p} = 3970, \rho_{b} = 997.1 , \alpha = 0.5, R = 10$, P = − 2.

**Figure 9:**
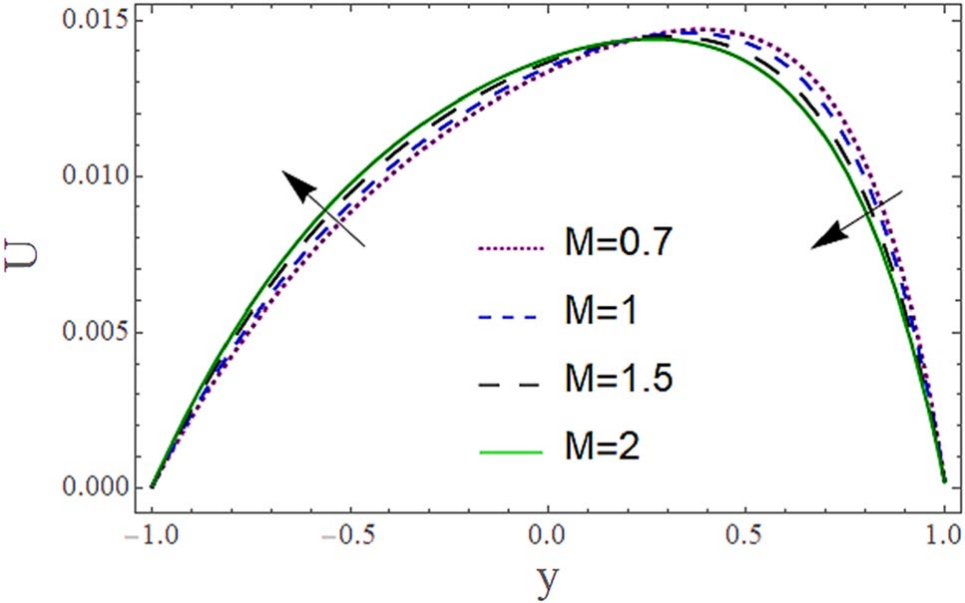
Behavior of $U{ }$ with “*y*” for distinct values of M for $W = 0.002, V = 0.05, {\o} = 0.1, \rho_{p} = 3970, \rho_{b} = 997.1 , \alpha = 0.5, R = 10$, P = − 2.

**Figure 10:**
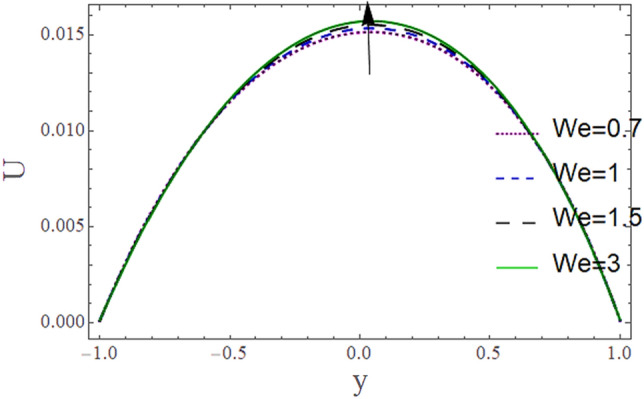
Behavior of $U{ }$ with “*y*” for distinct values of W for $M = 1, V = 0.05, {\o} = 0.1, \rho_{p} = 3970, \rho_{b} = 997.1 , \alpha = 0.5, R = 10$, P = − 2.

The^8^ has plotted graphs of u for $u = \frac{{\epsilon^{2} }}{2}\varphi_{20}^{{\prime}}$ only but we have ploted for Eq. (45). For graphing we have assumed that $u = U = \in \varphi_{1}^{{\prime}} e^{{i\alpha \left( {x - t} \right)}} + \frac{{\epsilon^{2} }}{2}\varphi_{20}^{{\prime}} .$ In Figs. 2, 3, 4, 5, 6 and 7 we have examined the change of velocity at the walls of a channel with the help of no-slip constants ${\text{E}}_{2}$ and ${\text{E}}_{3}$ by using porosity $V = 0.5,1,1.5,2$, permeability $W = 0.002,0.004,0.006,0.008$ and Eyring Powell parameter $M = 0.1,0.2,0.3,0.4$ and in Figs. 8, 9 and 10 we have checked the distribution of the velocity across the whole channel with porosity $V = 0.5,1,1.5,2$, permeability $W = 0.7,1,1.5,3$ and Eyring Powell parameter $M = 0.7,1,1.5,2$ In Addition, Figs. 14, 15 and 16 shows variations in temperature $\theta$ with y and t by keeping volume fraction ${\o}_{v} = 0.05,0.15,0.25,0.35 {\text{and porosity}} V = 5,10,15,20$.

In Figs. 2, 3 and 4, we have presented the Variation in $$E_{2}$$ with $$\alpha$$ for distinct values of *V, M* and *W*. Figure 2 reflects that the rise in the porosity of the boundary *V* rises the constant $$ E_{2}$$. The numerical result of Figs. 3 and 4 show that the constant $$E_{2}$$ falls with the rise in Eyring Powell Parameter *M* and the permeability parameter *W*.

In Figs. 5, 6 and 7, we have presented the Behavior of $E_{3}$ with $\alpha$ for distinct values of *V, M* and *W*. It is observed in Figs. 5 and 7 the constant $E_{3}$ falls with the rise in porosity *V* at the boundaries, and permeability parameter *W* while rises with the rise in the Eyring Powell Parameter *M*.

According to Figs. 2, 3, 4, 5, 6 and 7 fluid entering through the lower plate creates an injunction while fluid exiting through the upper plate creates a suction. Additionally, *M* is inversely proportional to the viscosity so by increasing viscosity, *M* decreases which means fluid becomes more viscous and hence velocity decreases. The value of constants $$E_{2}$$ become smaller and $$E_{3}$$ become larger as we increase the non-Newtonian viscoelastic parameter $$M$$ indicating that velocity rises as we rise the viscoelastic parameter overall but due to suction, the velocity of the upper portion of the channel drops so $$E_{2} $$ drops by rising $$M$$.

Figure 8 depicts how the mean flow drops in the lower segment and rises in the upper segment of the channel with a rise in the porosity of the walls. Figure 9 demonstrates that the mean velocity rises with the rise in the Eyring Powell fluid parameter $M$ in the lower portion while drops in the lower portion. Fluid behavior changes after the mean position $y = 0$ of the channel which shows in that segment injection is dominated over suction. Since $M \propto \frac{1}{\mu }$ which means when *M* is greater, fluid will be less viscous, and velocity will be greater and vice versa. According to this relation, overall velocity should have to rise in the whole channel by increasing M but in the upper portion, there is variation in behavior due to the impact of suction. According to Fig. 10 the mean velocity $U$ generally rises as permeability *W* rises means that more fluid can pass through pores with the rise on permeability.

**Figure 11:**
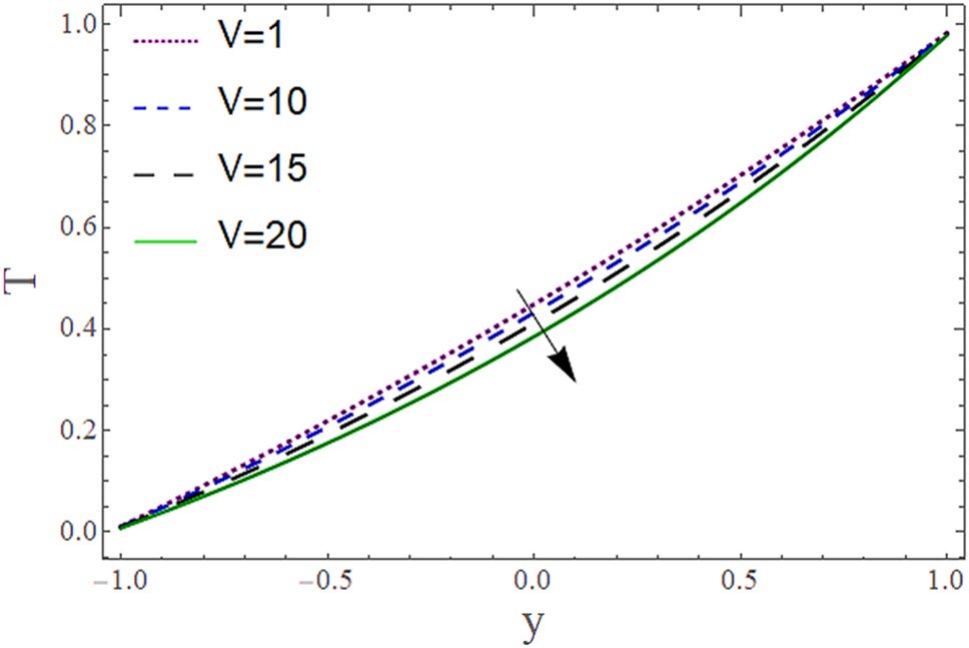
Behavior of $$T $$ with “*y*” for distinct values of V for $$R = 0.001, M = 1,$$ $$Pr = 6.9, M = 2, W = 0.2, P = - 3, \rho_{p} = 3970, \rho_{b} = 997.1, c_{p} = 880, c_{b} = 4200,$$ $$k_{p} = 32, k_{b} = 0.598, t = 0, x = 8.7, {\o}_{v} = 0.1, \alpha = 0.5, \varepsilon = 0.1$$

**Figure 12:**
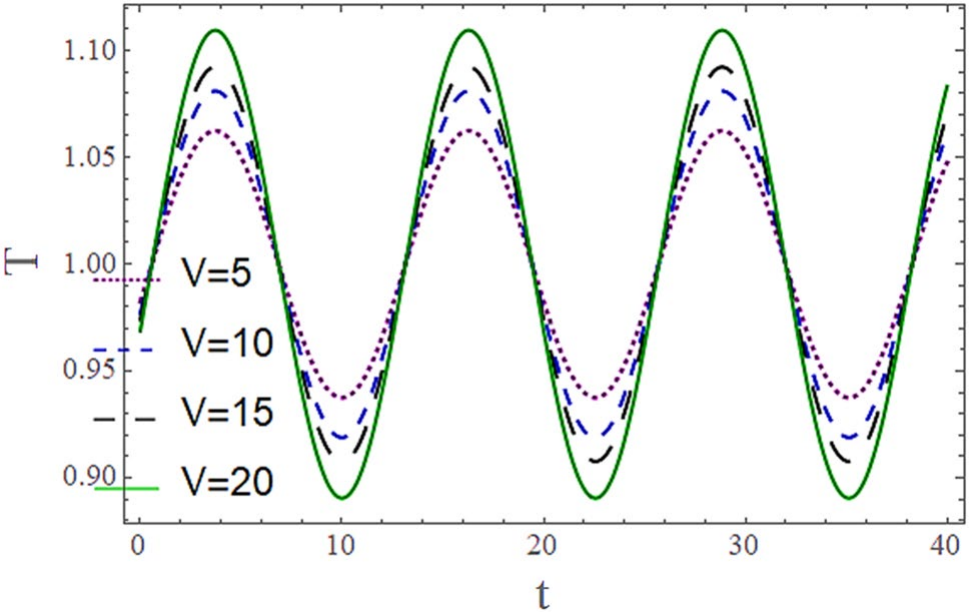
Behavior of $T{ }$ with “*t*” for distinct values of V for $R = 0.001, M = 1,$ $Pr = 6.9, M = 2, W = 0.2, P = - 3, \rho_{p} = 3970, \rho_{b} = 997.1, c_{p} = 880, c_{b} = 4200,$ $k_{p} = 32, k_{b} = 0.598, y = 1, x = 8.7, {\o}_{v} = 0.1, \alpha = 0.5, \varepsilon = 0.1$

**Figure 13:**
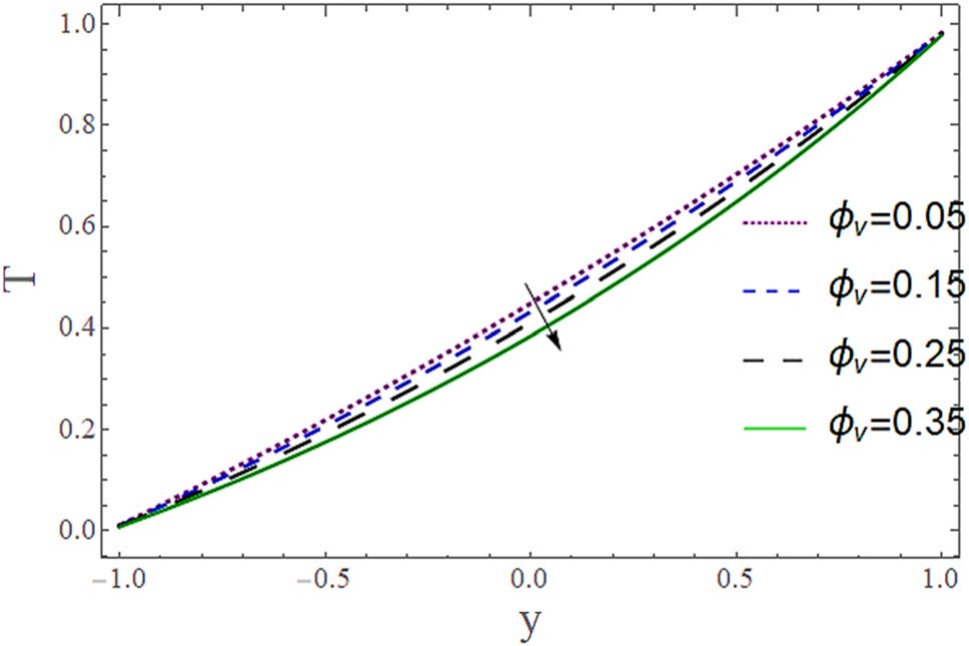
Behavior of $T{ }$ with “*t*” for distinct values of ${\o}_{v}$ for $R = 0.001, M = 1,$ $Pr = 6.9, M = 2, W = 0.2, P = - 3, \rho_{p} = 3970, \rho_{b} = 997.1, c_{p} = 880, c_{b} = 4200,$ $k_{p} = 32, k_{b} = 0.598, t = 0, x = 8.7, V = 5, \alpha = 0.5, \varepsilon = 0.1$

**Figure 14:**
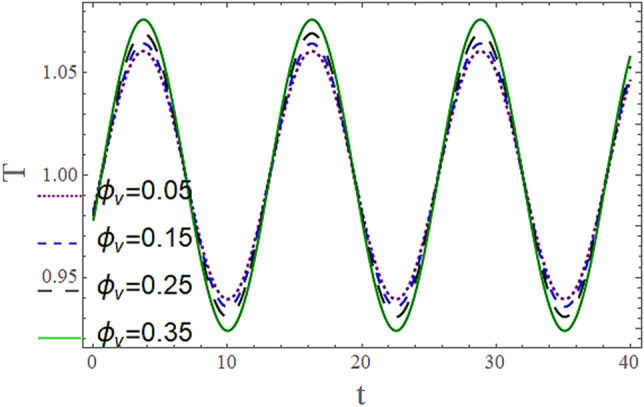
Behavior of $T{ }$ with “*t*” for distinct values of ${\o}_{v}$ for $R = 0.001, M = 1,$ $Pr = 6.9, M = 2, W = 0.2, P = - 3, \rho_{p} = 3970, \rho_{b} = 997.1, c_{p} = 880, c_{b} = 4200,$ $k_{p} = 32, k_{b} = 0.598, y = 1, x = 8.7, V = 5, \alpha = 0.5, \varepsilon = 0.1$

**Figure 15:**
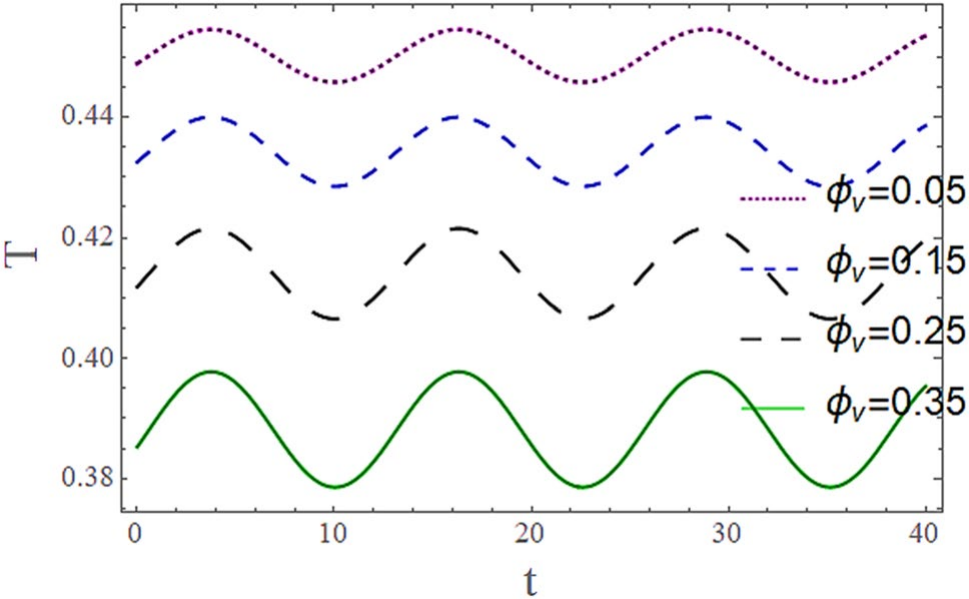
Behavior of $$T{ }$$ with “*t*” for distinct values of $${\o}_{v}$$ for $$R = 0.001, M = 1,$$$$Pr = 6.9, M = 2, W = 0.2, P = - 3, \rho_{p} = 3970, \rho_{b} = 997.1, c_{p} = 880, c_{b} = 4200,$$ $$k_{p} = 32, k_{b} = 0.598, y = 0, x = 8.7, V = 5, \alpha = 0.5, \varepsilon = 0.1$$

**Figure 16:**
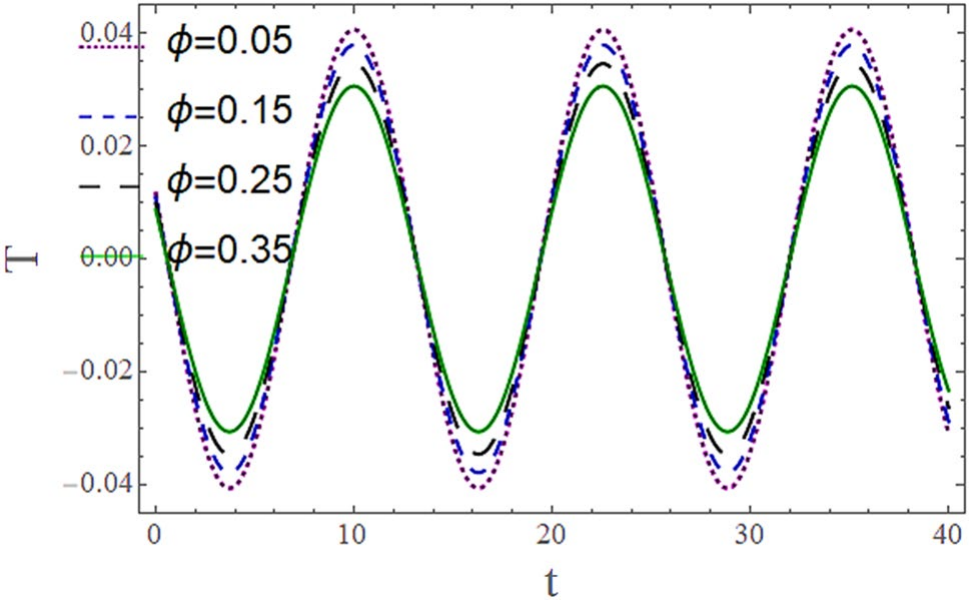
Behavior of $$T{ }$$ with “*t*” for distinct values of $${\o}_{v}$$ for $$ R = 0.001, M = 1,$$ $$Pr = 6.9, M = 2, W = 0.2, P = - 3, \rho_{p} = 3970, \rho_{b} = 997.1, c_{p} = 880, c_{b} = 4200,$$ $$k_{p} = 32, k_{b} = 0.598, y = - 1, x = 8.7, V = 5, \alpha = 0.5, \varepsilon = 0.1$$

 Figures 11 and 13 show the relation of temperature $$T$$ with Cartesian coordinate *y* and reflect that by increasing porosity *V* of the boundaries and volume fraction $${\o}_{v}$$ of Nanofluid $$T$$, temperature $$T$$ decreases which means when more fluid is entering the channel it decreases the temperature of the fluid present in the channel. Also, when there is a larger quantity or volume of nanoparticles it decreases temperature which also shows that here nanoparticles act as coolants. In Fig. 15 it is demonstrated that by increasing the Reynolds number $$R$$ temperature $$T$$ increases. Figures 12, 14, 15 and 16 show the relation of temperature $$T $$ with time t and demonstrate that by increasing porosity *V* of the boundary’s temperature $$T$$ decreases overall and volume fraction $${\o}_{v}$$ of Nanofluid increases at upper extreme and decreases at lower extreme and other portions of channel. It should have to decerease overall but at upper extreme variation is due to suction and show sinusoidal behavior after some time due to peristaltic boundaries. At $$y = 1$$, temperature is in between 1 $$\pm \delta$$ then going towards other boundary it starts decreasing and become $$0 \pm \delta$$ at $$y = - 1$$.

**Figure 17:**
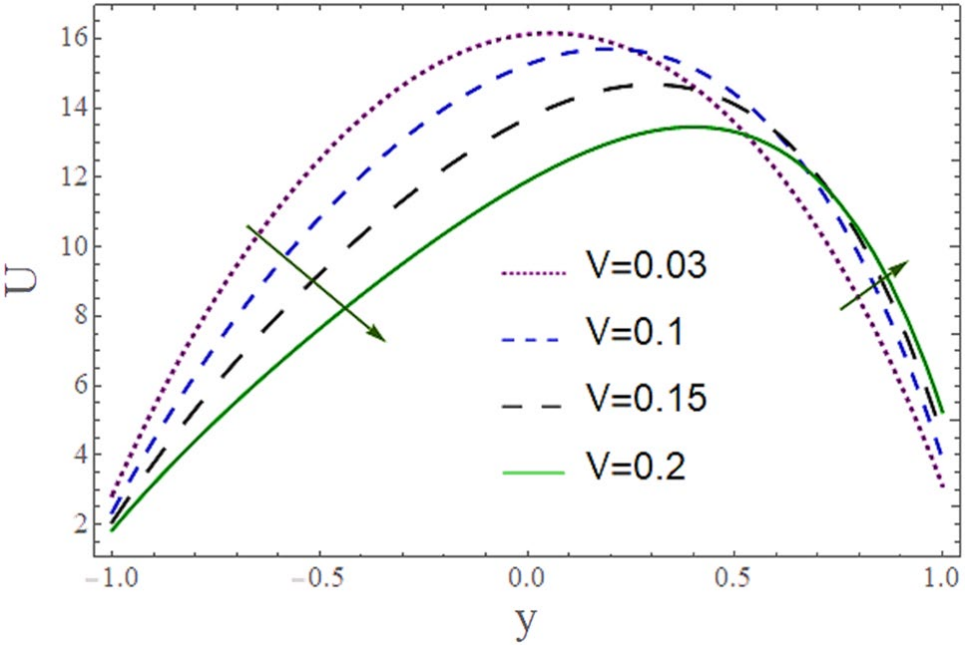
Behavior of $U{ }$ with “*y*” for distinct values of V for W = 0.002, M = 0, ${\text{\o}}$ = 0, $\rho_{p} = 3970$, ${ }\rho_{b}$ = 997.1, $\alpha = 0.5,{\text{ R}} = 10,$

**Figure 18:**
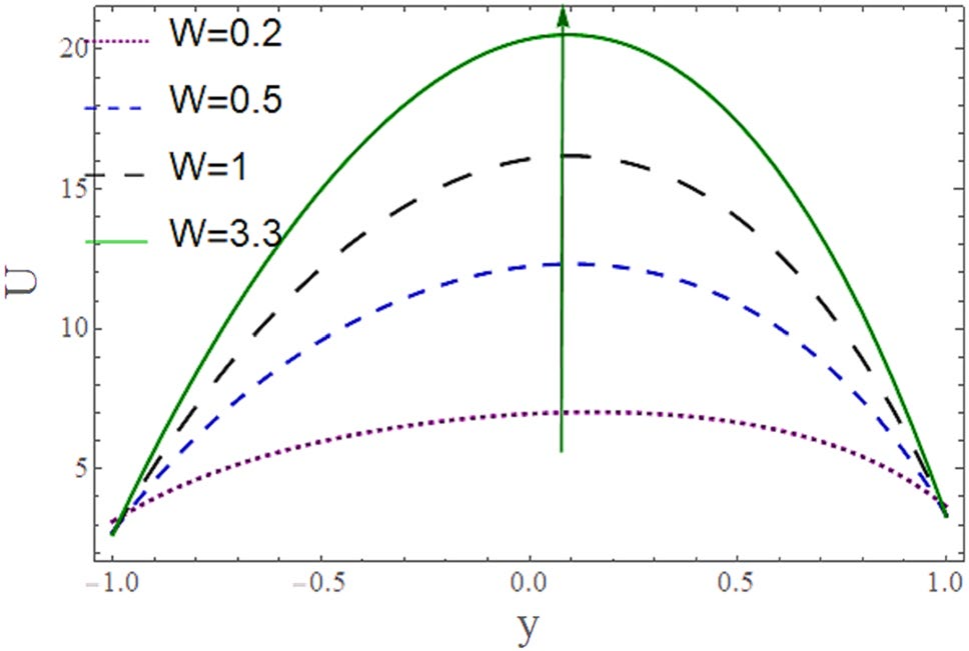
Behavior of $U{ }$ with “*y*” for distinct values of W for M = 0, V = 0.05, ${\text{\o}}$ = 0, $\rho_{p} = 3970$, ${ }\rho_{b}$ = 997.1, $\alpha = 0.5$, R = 10.

In Figs. 17 and 18 the results of Husseny and Shehawey have been reserved which means by zeroing the non-Newtonian effects and volume fraction of the Nanofluid the effects which have been analyzed for the Newtonian case have been conserved. These two figures are drawn by keeping $U = \varphi_{20}^{{\prime}}$ (for only 2nd order perturbation solution only).

## Conclusions

In this study, we have talked about an Eyring Powell fluid model with $\text{Al}_{2} \text{O}_{3}$ as nanoparticle in this investigation. We draw the following conclusions regarding our free pumping case:

- The dominant constant $E_{2}$ in mean velocity for upper wall rises with a rise in porosity *V*, while $E_{2}$ falls with the rise in Eyring Powell parameter *M* and Permeability *W*.
- The constant $E_{3}$ falls with the rise in Permeability *W* and porosity *V* while rises with the rise in the Eyring Powell parameter *M*.
- The Velocity drops with the rise in the viscosity but in the upper segment of channel suction disturbs this behavior.
- The rise in permeability raises the velocity of the flow means it allows more fluid to pass through the medium.
- The Velocity rises with the rise in the Eyring Powell parameter in lower segment of channel and drops in upper segment of the channel.
- The rise in the porosity of the wall causes the drop in the mean velocity of the lower segment of the channel and rises in the upper segment of the channel.
- Temperature $T$ drops with the rise in porosity $V$.
- The rise in the volume fraction ${\o}_{v}$ of Nanofluid drops the temperature.

Data availability

The authors states that all the files are provided in the paper no hidden file is required however if journal required any further data from us we will provide and the corresponding author is responsible to provide to the journal.

## List of symbols

*x*: Cartesian coordinate in the direction of wave propagation and normal of the wall
*u,v*: Velocity component in direction of x and y
***V***: Velocity vector
$$\rho$$: Density
$\rho_{nf}$: Effective density of nanofluid
$\rho_{p}$: Density of nanoparticle $\text{Al}_{2} \text{O}_{3}$
$\rho_{b}$: Density of water
$$\mu$$: Viscosity
$\mu_{nf}$: Effective viscosity of nanofluid
$$\mu_{p}$$: Viscosity of $$\text{Al}_{2} \text{O}_{3}$$
$\mu_{b}$: Viscosity of water
*k*: Thermal conductivity
$k_{nf}$: Nano fluid’s effective thermal conductivity
$k_{p}$: Thermal conductivity of nanoparticle $\text{Al}_{2} \text{O}_{3}$
$k_{b}$: Thermal conductivity of water
$c_{p}$: Specific heat capacity
$c_{{p_{nf} }}$: Nano fluid’s specific heat capacity
$c_{p}$: Specific heat capacity of $\text{Al}_{2} \text{O}_{3}$
$c_{b}$: Specific heat capacity of water
*p*: Pressure
$\beta ,c_{1}$: Eyring fluid constants
*W*: Permeability
*V*: Suction/injection velocity
*T*: Temperature
$$\psi$$: Stream function
*M*: Eyring fluid parameter
*R*: Reynolds number
*Pr*: Prandtl number
$\delta$: Sinusoidal vertical displacement of the wall
$$\lambda$$: Wavelength
*a*: Amplitude
*c*: Wave speed
*b*: Uniform thickness of half channel from the centre line
$${\o}_{v}$$: Volume fraction
$$\alpha$$: Wave number
$$\varepsilon$$: Amplitude ratio
